# Feshbach resonances in the F + CHD_3_ → HF + CD_3_ reaction†

**DOI:** 10.1039/d3sc02629a

**Published:** 2023-06-24

**Authors:** Shu Liu, Jun Chen, Xiaoren Zhang, Dong H. Zhang

**Affiliations:** a State Key Laboratory of Molecular Reaction Dynamics, Dalian Institute of Chemical Physics, Chinese Academy of Sciences Dalian Liaoning 116023 China liushu1985@dicp.ac.cn zhangdh@dicp.ac.cn; b University of Chinese Academy of Sciences Beijing 100049 China; c State Key Laboratory of Structure Chemistry, Fujian Institute of Research on the Structure of Matter, Chinese Academy of Sciences Fuzhou Fujian 350002 China

## Abstract

The signature of dynamics resonances was observed in the benchmark polyatomic F + CH_4_/CHD_3_ reactions more than a decade ago; however, the dynamical origin of the resonances is still not clear due to the lack of reliable quantum dynamics studies on accurate potential energy surfaces. Here, we report a six-dimensional state-to-state quantum dynamics study on the F + CHD_3_ → HF + CD_3_ reaction on a highly accurate potential energy surface. Pronounced oscillatory structures are observed in the total and product rovibrational-state-resolved reaction probabilities. Detailed analysis reveals that these oscillating features originate from the Feshbach resonance states trapped in the peculiar well on the HF(*v*′ = 3)–CD_3_ vibrationally adiabatic potential caused by HF chemical bond softening. Most of the resonance structures on the reaction probabilities are washed out in the well converged integral cross sections (ICS), leaving only one distinct peak at low collision energy. The calculated HF vibrational state-resolved ICS for CD_3_(*v* = 0) agrees quantitatively with the experimental results, especially the branching ratio, but the theoretical CD_3_ umbrella vibration state distribution is found to be much hotter than the experiment.

## Abstract 2

The F + CHD_3_ reaction possesses Feshbach resonances trapped in the chemical bond softening well on the HF(*v*′ = 3)–CD_3_ VAP, which prove that the hydrogen abstraction reactions involving a F atom share the same resonance picture.

## Graphical abstract

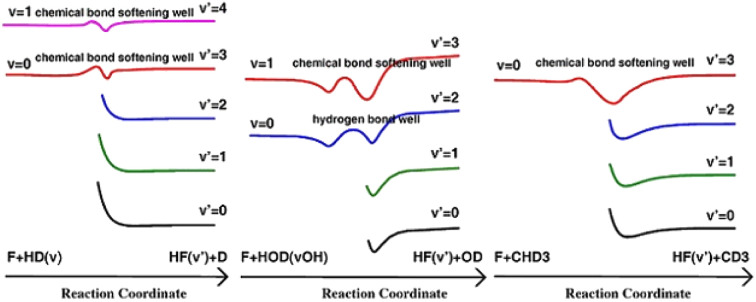

Over the past decades, a clear physical picture of the reaction resonances in the F + H_2_/HD/H_2_O/HOD/NH_3_ reactions has been established through close interaction between high-resolution crossed-molecular beams or transition-state spectroscopy experiments and quantum dynamics calculations on highly accurate potential energy surfaces (PESs).^1–10^ All these hydrogen abstraction reactions involving a F atom possess similar dynamical resonances trapped by the HF vibrational excited adiabatic potential wells in the post-barrier region.

Like the F + H_2_/H_2_O/NH_3_ systems, the F + CH_4_ → HF + CH_3_ reaction is another benchmark system for highly exothermic hydrogen abstraction reactions with a low and early barrier. It has a bent transition-state geometry, a shallow van der Waals (vdW) well in the entrance channel and a deeper vdW well in the exit channel, both with linear configurations. The reaction plays an important role in atmospheric chemistry and can yield a highly inverted HF(*v*′) vibrational distribution, which shows great potential in the production of chemical lasers.^11–13^ Liu and coworkers reported the first experimental signature of reactive resonances in a polyatomic reaction in the F + CH_4_/CHD_3_ reaction.^14–17^ In the F + CHD_3_(*v* = 0) reaction, they observed a step-like feature in the excitation function of the CD_3_(*v* = 0) + HF channel at low collision energies,^15^ which echoes the resonance fingerprints in the integral cross sections (ICS) of the F + HD → HF + D reaction.^18,19^ Furthermore, the excitation function of the CHD_2_(*v* = 0) + DF channel displays a ‘kink’ in shape at about the same energy.^15^ The differential cross-sections (DCS) of the two isotopic channels both show a near-threshold ridge and oscillatory forward–backward peaking, which also provide strong support for reactive resonances.^16^ The cross-sections of the bending-excited reaction F + CHD_3_(*v*_b_ = 1) also show peak features for the CD_3_(*v*_2_ = 0,1) + HF(*v*′ = 3) channels.^17^ In addition, they observed anticorrelation in the vibrational states of the coincident product pairs of CD_3_(*v*_2_) and DF(*v*′) in the F + CD_4_ reaction.^20–22^

Theoretically, great efforts have been devoted to the construction of an accurate potential energy surface (PES) for the F + CH_4_ system.^23–28^ In 2009, Czakó *et al.* constructed a full-dimensional PES with permutational invariant polynomials (PIP) method called CSBB PES,^29^ and subsequently improved the PES by including the spin–orbit (SO) coupling term of the F atom.^30^ In 2014, Westermann *et al.*^31,32^ constructed the coupled diabatic WEM PESs for the entrance channel region containing vibronic as well as SO coupling effects and connected the lowest adiabatic one with the CSBB PES in the interaction region to acquire a global adiabatic PES called PWEM PES. Later, they constructed the globally defined coupled diabatic PESs^33^ by using the same set of diabatic electronic states in the transition state region and all four exit channels. In 2018, our group constructed a global PES for the system using a neural network (NN) fitting method based on 99 000 UCCSD(T)-F12a/aug-cc-pVTZ *ab initio* energies.^34^ Correction terms considering the influence of a larger basis set as well as spin–orbit couplings were further implemented with a hierarchical scheme. With a fitting error of ∼4.87 meV for energies within 2.5 eV, our NN PES is substantially more accurate than the previous ones.

The theoretical evidence for reactive resonances in the F + CH_4_ reaction was first reported by Nyman *et al.* through the oscillations in the cumulative reaction probabilities (CRP) and the dramatic change of DCS with collision energy^35^ by using the time-independent three-dimensional rotating line umbrella (RLU) quantum scattering model. Wang showed the resonance features in the initial-state-selected total reaction probabilities and the ICS calculated by using the four-degree-of-freedom quantum dynamics approach on the CSBB PES.^36^ Westermann *et al.* found resonances trapped in the entrance channel vdW well of the F + CH_4_ system^32^*via* the transition-state spectra calculated using the full-dimensional (12D) multi-configurational time-dependent Hartree (MCTDH) approach on the WEM PESs. For the F + CHD_3_ reaction, von Horsten and Clary studied the reactive resonances using a two-dimensional model, which only described the bond breaking and bond forming explicitly. The final state resolved ICS for both reaction channels showed qualitative agreement with experimental results, although discrepancies remained in the branching ratios. They attributed the low energy peaks in the CRP to quasi-bound states with a quantum number *v*′ = 3 of the H–F stretch mode, and *v*′ = 4 of the D–F stretch mode in the product region.^37^ Qi *et al.* performed eight-dimensional (8D) wave packet dynamics calculations^38^ on the PWEM PES, and Zhao *et al.* performed seven-dimensional (7D) non-adiabatic wave packet dynamics calculations^39^ on the global vibronically and SO-coupled diabatic PESs for the F + CHD_3_ reaction. They both reported fast-oscillating structures on the total reaction probabilities at low collision energies, but attributed them to the resonances in the pre-reaction vdW well. Therefore, the origin of the resonances in the reaction is still not clear.

Here, we report six-dimensional (6D) state-to-state quantum dynamics studies of the F + CHD_3_ → HF + CD_3_ reaction on the NN PES. Pronounced oscillatory structures are observed in the total reaction probabilities, in particular at collision energies below 0.05 eV. Detailed analysis reveals that these oscillating features originate from the Feshbach resonance states trapped in the peculiar well on the HF(*v*′ = 3)–CD_3_ vibrational adiabatic potential (VAP) caused by HF bond softening, mainly producing HF(*v*′ = 2). The calculated vibrational state-specific excitation functions of HF(*v*′) + CD_3_(*v* = 0) are in good agreement with the experimental results. However, the theoretical CD_3_ umbrella vibration state distribution is found to be much hotter than the experiment.

The reduced dimensional model employed in the time-dependent wave packet (TDWP) calculations here was the free-torsion seven-dimensional (FT-7D) model we proposed for the X + YCZ_3_ → XY + CZ_3_ reaction in 2013, by assuming that the CZ_3_ group can rotate freely with respect to its *C*_3v_ symmetry axis.^40^ This is an additional approximation introduced to the original eight-dimensional (8D) model of Palma and Clary with the nonreacting CZ_3_ group constrained in the *C*_3v_ symmetry^41^ to reduce computational costs. With the bond length of CD fixed at its equilibrium value in the reactant (2.06 bohr) because it essentially does not change during the reaction, the number of degrees of freedom included in the calculation was reduced to six. We used the multiple-step reactant-product decoupling (MRPD) method^42–44^ to obtain the state-to-state information. We carried out state-to-state calculations for the total angular momentum *J*_tot_ = 0, 10, 15, 20, 25, 30, 40, 60, and 80 to converge the ICS for collision energies up to 0.3 eV. For details of the numerical parameters, please refer to the ESI.†

Fig. 1(A) shows the total reaction probabilities for the total angular momentum *J*_tot_ = 0 calculated using the 7D (with the CH bond length fixed in the original 8D model) and FT-6D models. The converged reaction probabilities rise rapidly as the collision energy increases from 0 to 0.2 eV without a reaction threshold, exhibiting many sharp and narrow oscillatory structures, especially for collision energies below 0.05 eV. Except at very low collision energies, the present reaction probabilities on the NN PES are larger than those^38,39^ on the PWEM and vibronically and SO-coupled diabatic PESs. It can be seen that the 6D treatment can produce similar reaction probabilities to the 7D results, except that the 6D probabilities are slightly smaller between 0.02 and 0.15 eV. We, therefore, used the 6D model to carry out state-to-state calculations to reduce computational costs.

**Fig. 1:**
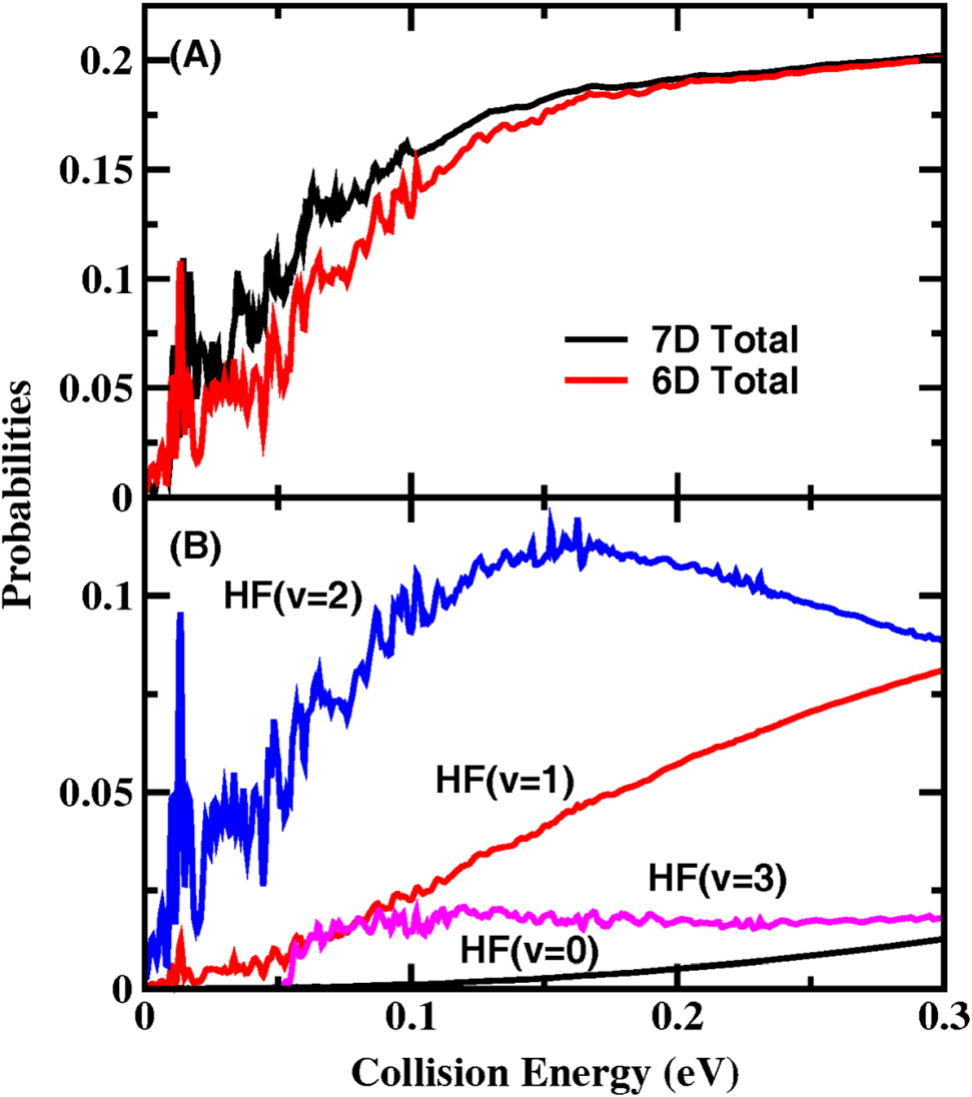
(A) Total and (B) product HF vibrational state-resolved *J*_tot_ = 0 reaction probabilities for the initial ground rovibrational state as a function of collision energy.

Fig. 1(B) shows the 6D HF vibrational state-resolved reaction probabilities as a function of collision energy. The HF(*v*′ = 2) state has the largest population in the entire energy region considered here, indicating an inverted HF vibrational state distribution. With increasing collision energy, the reaction probabilities for the HF(*v*′ = 1) state gradually increase, and the fraction of HF(*v*′ = 2) decreases after 0.16 eV. The lowest vibrational state HF(*v*′ = 0) can only be observed at *E*_c_ > 0.1 eV as a smooth curve. The HF(*v*′ = 3) channel opens at a collision energy of 0.05 eV and has a probability that remains almost constant at high collision energies. The probabilities of the HF(*v*′ = 1, 2, 3) states all show oscillation peaks at low collision energies and become smooth in the high-energy region.

To investigate the dynamical origin of the oscillatory structures, we calculated the *J*_tot_ = 0 time-independent (TID) scattering wavefunctions at some collision energies for the reaction by performing Fourier transform of the time-dependent wavefunctions. Inspection of the two-dimensional contour of the wavefunction for the collision energy at 0.0135 eV in the product coordinates shown in Fig. 2(A) reveals that there exist three nodes along the H–F coordinate (correlating to the HF product) in the HF–CD_3_ complex, but only two nodes for the outgoing wavefunction with a sudden decrease in amplitude. The sudden change in the nodal structure and amplitude of the wavefunction is the characteristic feature of a Feshbach resonance, indicating that the resonance is trapped in the HF(*v*′ = 3) VAP well and produces mainly the HF(*v*′ = 2) product. Similar to the F + H_2_/HD/HOD(*v* = 1) reactions, the resonance wavefunctions of the title reaction are also narrow and located close to the barrier, and do not traverse the entire vdW well. The TID wavefunctions at the other low collision energies are found to have the same nodal structure as shown in Fig. 2(A).

**Fig. 2:**
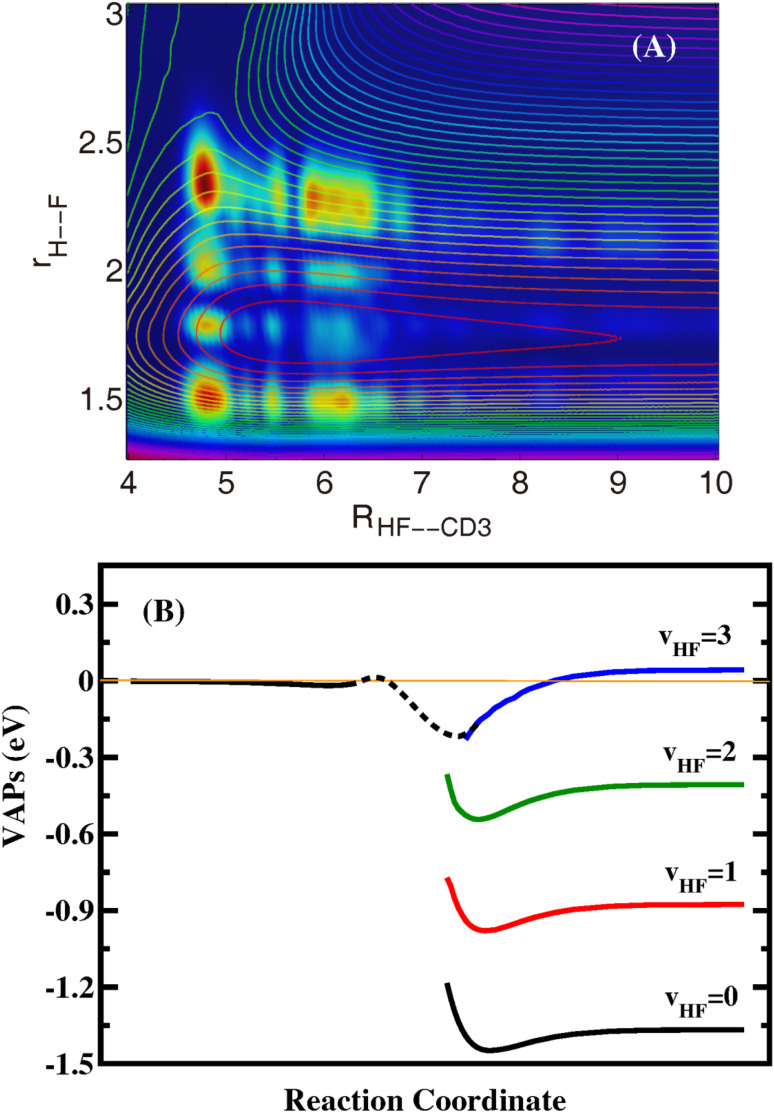
(A) Reactive scattering wavefunctions for the title reaction in the two Jacobi coordinates *R*(HF–CD_3_) and *r*(H–F) with other coordinates integrated at a collision energy of 0.0135 eV. The contour lines are the corresponding 2D PESs along the two reactive bonds *R*(HF–CD_3_) and *r*(H–F) with the other coordinates optimized. (B) The calculated VAPs for the F + CHD_3_ → HF + CD_3_ reaction with different vibrational states of HF.

To further confirm the mechanism of the resonance, we calculated the VAP for HF(*v*′ = 0–3) in the product region. The reaction coordinates are calculated in terms of *R*_F–CHD_3__ on the reactant side and *R*_HF–CD_3__ on the product side. As can be seen in Fig. 2(B), the VAP curves relevant to the vibrational states HF(*v*′ = 0–2) only have vdW wells. The minimum position of the vdW well moves from *R*_HF–CD_3__ = 5.7 *a*_0_ for *v*′ = 0, to 5.6 *a*_0_ for *v*′ = 1, and 5.4 *a*_0_ for *v*′ = 2, while the well depth increases slowly with *v*′, from 0.082 eV for *v*′ = 0, to 0.103 eV for *v*′ = 1, and 0.137 for *v*′ = 2. On the other hand, the HF(*v*′ = 3)–CD_3_ VAP well is substantially deeper and is located much closer to the barrier than the HF(*v*′ = 0–2)–CD_3_ vdW wells, which is the very peculiar well caused by chemical bond softening. The HF(*v*′ = 3)–CD_3_ VAP has an asymptotic value of 0.0472 eV measured from the ground state of the reactants, and a well bottom lower than the ground state of the reactants. This means that the F + CHD_3_ reaction can access the Feshbach resonances supported by the HF(*v*′ = 3)–CD_3_ VAP well even with zero collision energy, which can only decay into lower HF(*v*′ = 0–2) vibrational states *via* vibrational predissociation before the HF(*v*′ = 3) channel opens. Therefore, the oscillating structures on the HF(*v*′ = 1,2) probabilities in Fig. 1(B) originate from the Feshbach resonance states trapped the peculiar HF(*v*′ = 3)–CD_3_ VAP well created by the HF bond softening. The discernible oscillating structures on the HF(*v*′ = 3) probabilities and HF(*v*′ = 2) probabilities for *E*_c_ > 0.05 eV may originate from the bending excited HF(*v*′ = 3)–CD_3_ VAP supported resonance states.

To investigate the effect of the resonance structures on the ICS, we calculated the ICS for the title reaction based on the reaction probabilities for *J*_tot_ = 0, 10, 15, 20, 25, 30, 40, 60 and 80, as shown in Fig. S1.† With increasing *J*_tot_, the reaction probability curve shifts to higher energy, and the influence of the resonances on the reaction gradually fades. In order to take into account the change in the resonance structures with collision energy and *J*_tot_, we estimated the reaction probabilities for partial waves *J*_1_ < *J* < *J*_2_ as*P*^*J*^(*E*) = *P*_*J*_1__^*J*^(*E*) × *f* cos(*J*) + *P*_*J*_2__^*J*^(*E*) × [1 − *f* cos(*J*)],where
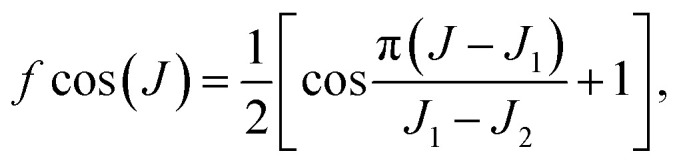


*P*_*J*_1__^*J*^(*E*) = *P*^*J*_1_^(*E* − Δ*E*_1_) and *P*_*J*_2__^*J*^(*E*) = *P*^*J*_2_^(*E* + Δ*E*_2_) are obtained using the *J*-shifting approximation with Δ*E*_1_ = [*J*(*J* + 1) − *J*_1_(*J*_1_ + 1)] × *B*, Δ*E*_2_ = [*J*_2_(*J*_2_ + 1) − *J*(*J* + 1)] × *B*. *B* is a varying rotational constant for different [*J*_1_, *J*_2_] intervals, which is obtained by minimizing the difference between *P*^*J*_1_^(*E* − [*J*_2_(*J*_2_ + 1) − *J*_1_(*J*_1_ + 1)] × *B*) and *P*^*J*_2_^(*E*). Fig. 3(A) shows the HF vibrational state-resolved ICS as a function of collision energy. The shapes of the excitation function are similar to the *J*_tot_ = 0 reaction probabilities in Fig. 1(B), except that the ICS of HF(*v*′ = 3) are higher than HF(*v*′ = 1) above *E*_c_ = 0.062 eV. Most resonance structures on the reaction probabilities vanish in the ICS after averaging over impact parameter *J*_tot_, leaving only one distinct peak near *E*_c_ = 0.015 eV. In order to test the convergence of the ICS, four reaction probabilities (*J*_tot_ = 10, 15, 25, 30) were removed, and the cross-sections obtained using the 5 *J*_tot_ were in good agreement with those calculated with all 9 *J*_tot_, even the resonance peaks, as shown in Fig. S2.† This proves that the *J*-shifting approximation method used here is reliable for reproducing the resonance structures on the reaction probabilities. Fig. S3† shows the normalized HF vibrational populations as a function of collision energy for the title reaction, which provide a comparison to the quasi-classical trajectories (QCT) results obtained using different methods on the CSBB PES.^45^ It can be seen that the GB_ZPVE_ method can reproduce the results near the threshold very well, while the GB_all_ method is more suitable for higher energies.

**Fig. 3:**
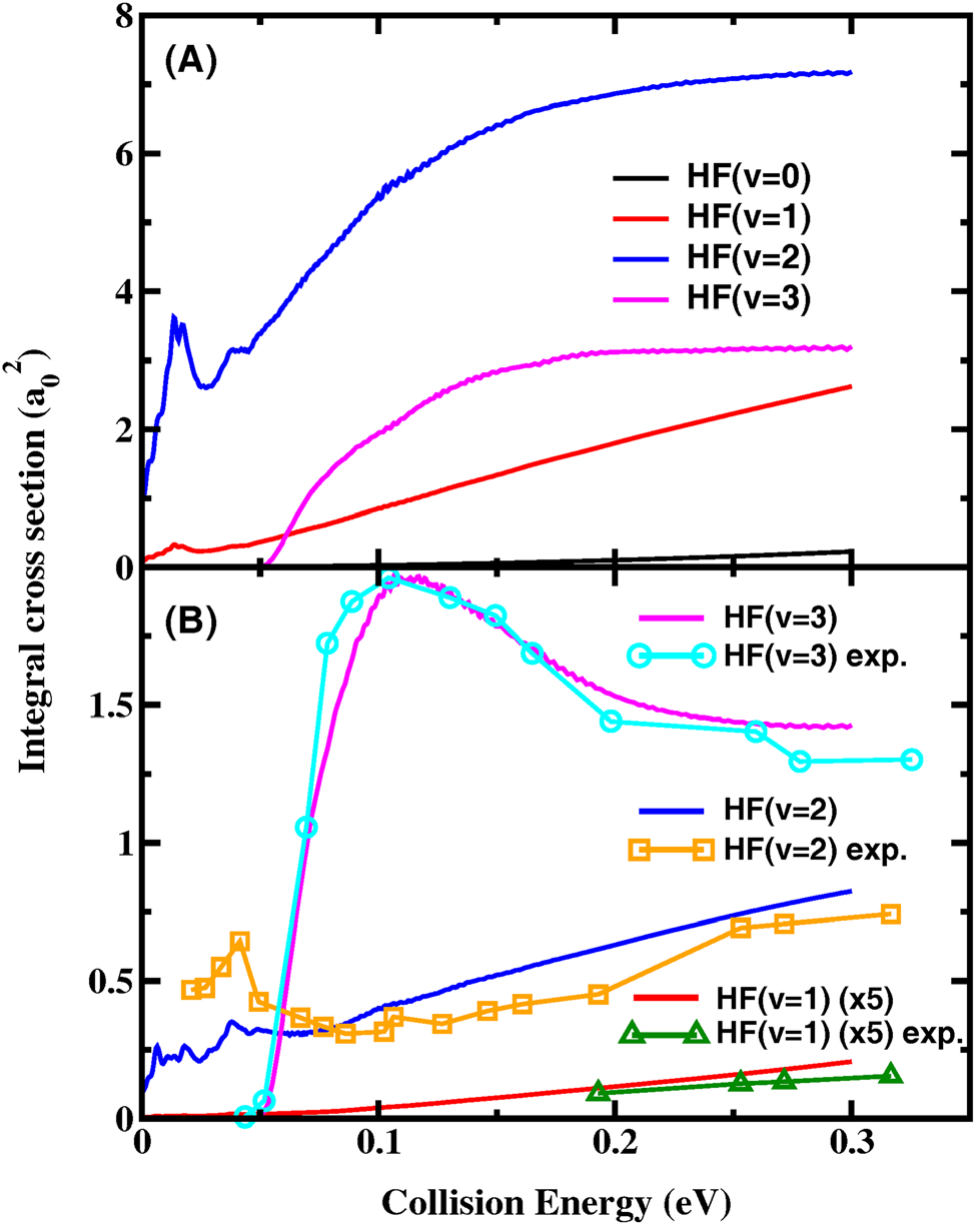
Product HF vibrational state-resolved ICS for the title reaction as a function of collision energy (A) including all the rovibrational states of CD_3_ and (B) for CD_3_(*v* = 0) together with the experimental excitation functions in ref. 15.

Fig. 3(B) shows the HF vibrational state-resolved ICS for CD_3_(*v* = 0) as a function of collision energy, along with the experimental excitation functions in ref. 15. The experimental and theoretical vibrational branching ratios of the product HF are in quantitative agreement with each other. The HF(*v*′ = 2) state is the sole contributor to the ICS in the low-energy regime. After the HF(*v*′ = 3) channel opens at *E*_c_ = ∼0.05 eV, its ICS rises rapidly and becomes the dominant channel. After reaching its peak at 0.1 eV, the HF(*v*′ = 3) ICS declines with further increase in the collision energy, causing the relative branching ratio of (*v*′ = 3)/(*v*′ = 2) to decrease. Both the experimental and theoretical ICS of the HF(*v*′ = 1) state are very small throughout the entire energy region considered here. The experimental excitation functions of HF(*v*′ = 2) have a step-like feature near 0.04 eV as evidence of the resonance. Our theoretical ICS of the HF(*v*′ = 2) state shows a peak at about the same energy, but with a lower amplitude.

As can be seen, the theoretical ICS including all the rovibrational states of CD_3_ shown in Fig. 3(A) are very different from those shown in Fig. 3(B) for the vibrational ground state of CD_3_, indicating that the HF vibrational distributions strongly depend on the CD_3_ umbrella states, or there exists a strong correlation between the vibrational distributions of the two product molecules. Fig. 4 shows the correlated ICS of the HF(*v*′) + CD_3_(*v*_2_) product pair at collision energies of 0.0145 eV, 0.1 eV, 0.2 eV and 0.3 eV. The branching ratios are obtained by normalizing the CD_3_ umbrella vibration distribution for each HF state at a given *E*_c_. A strong anti-correlation between the vibration excitations of the paired products is apparent. At all four collision energies, as the HF excitation increases, the umbrella mode excitations of the correlated CD_3_ show a noticeable decrease, and the proportion of CD_3_(*v*_2_ = 0) becomes increasingly larger. The CD_3_ umbrella state with the largest population changes from *v*_2_ = 6 for *v*′ = 0, to *v*_2_ = 4 for *v*′ = 1, to *v*_2_ = 3 for *v*′ = 2, and to *v*_2_ = 0 for *v*′ = 3. This strong anti-correlation between HF stretching and CD_3_ umbrella motion is in agreement with what Liu and coworkers observed in the F + CD_4_ reaction,^20–22^ but the theoretical CD_3_ umbrella mode excitations are much hotter than those observed in the F + CD_4_ experiment, which were mainly populated in *v*_2_ = 0–3. Because the umbrella angle of CD_3_ in the transition state and that in the product are quite different for this early barrier reaction, strong vibrational excitation for umbrella motion is expected. The QCT-correlated ICS for HF(*v*′) + CD_3_(*v*_2_) at a collision energy of 2.8 kcal mol^−1^ (0.121 eV) in ref. 45 showed the same anti-correlation trend, except that the present quantum mechanical (QM) CD_3_ excitation is slightly hotter for HF(*v*′ = 2) and slightly colder for HF(*v*′ = 3), as shown in Fig. S4.†

**Fig. 4:**
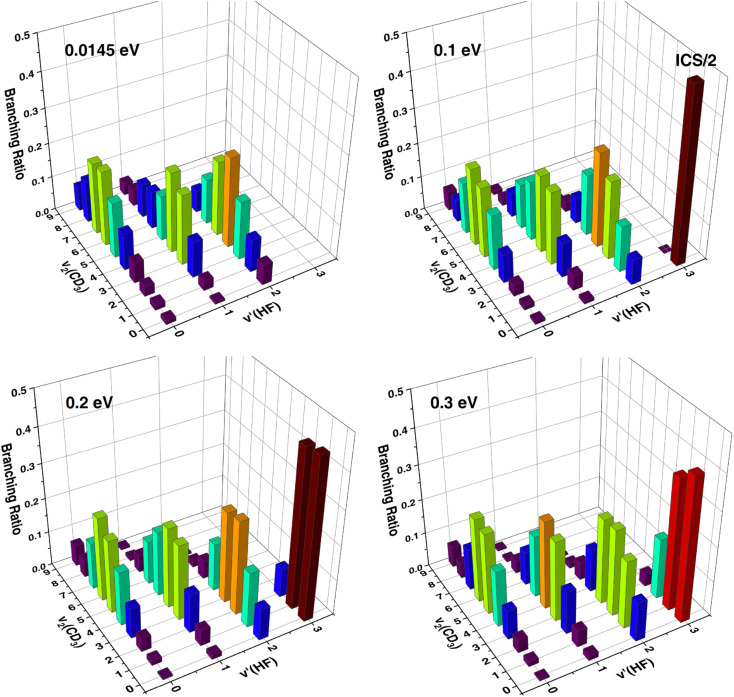
Three-dimensional representation of the correlated ICS of the product pair of HF(*v*′) and CD_3_(*v*_2_) at collision energies of 0.0145 eV, 0.1 eV, 0.2 eV and 0.3 eV; the sum of correlated CD_3_ umbrella vibration populations for each HF state at a given *E*_c_ are scaled to unity.

Fig. S5† shows the correlated ICS of the HF(*v*′) + CD_3_(*v*_2_) product pair, but the sum of the HF vibration populations for each CD_3_ state at a given *E*_c_ is scaled to unity. For the ground CD_3_ state, the HF vibration distribution is highly inverted, all peaking at *v*′ = 3, except that the HF(*v*′ = 3) channel is energetically closed at *E*_c_ = 0.0145 eV. With higher umbrella excitation of CD_3_, the vibration distribution of the correlated HF becomes colder, peaking at *v*′ = 2 for *v*_2_ = 1–6 and at *v*′ = 1 for *v*_2_ = 7–9 (for *E*_c_ = 0.1 eV), at *v*′ = 2 for *v*_2_ = 2–5 and at *v*′ = 1 for *v*_2_ = 6–9 (for *E*_c_ = 0.2 eV), and at *v*′ = 2 for *v*_2_ = 1–4 and at *v*′ = 1 for *v*_2_ = 5–9 (for *E*_c_ = 0.3 eV).

The anticorrelation of the HF(*v*′) + CD_3_(*v*_2_) products in terms of the energy distribution is shown in Table S1 and S2.† At a fixed *E*_c_, the average vibration energy of CD_3_ (〈*E*_*v*_2__〉_CD_3__) decreases with higher vibrational excitation of the correlated HF, while 〈*E*_*v*_〉_HF_ decreases when more energy is deposited into the umbrella mode of the CD_3_ coproduct. Because the vibrational energy levels of HF are wide enough to offset the decrease in the correlated average vibration energy of CD_3_, the sums of 〈*E*_*v*_2__〉_CD_3__ + *E*_HF_(*v*′) increase with *v*′. On the other hand, the trend in 〈*E*_*v*_〉 _HF_ + *E*_CD_3__(*v*_2_) is related to both *v*_2_ and *E*_c_.

Therefore, our quantum dynamics study of the F + CHD_3_ → HF + CD_3_ reaction on the highly accurate NN PES revealed that there exist pronounced oscillating structures in the total reaction probabilities, in particular in the low-collision-energy region, due to dynamical resonances trapped in the peculiar HF(*v*′ = 3) well in the post-barrier region, similar to those in the F + H_2_/HD/H_2_O/HOD reactions. Most of the resonance structures on the reaction probabilities are washed out in the ICS, leaving only a few oscillating structures in the ICS at low collision energies, in agreement with experimental observation. The theoretical HF vibrational state-resolved excitation functions for CD_3_(*v* = 0) agree very well with the experimental results, indicating that both the NN PES and quantum-dynamics method are sufficiently accurate for this reaction. A strong anti-correlated excitation of the two product vibrators HF(*v*′) and CD_3_(*v*_2_) was found in the reaction, similar to what was observed experimentally, but the theoretical CD_3_ umbrella vibration state distribution is much hotter than those observed in the F + CD_4_ experiment.

## Data availability

The data that support the findings of this study are available from the corresponding author upon request.

## Author contributions

S. L. and D. H. Z. conceived the research and wrote the manuscript. S. L. performed the quantum dynamical calculation. J. C. and X. Z. developed the PES.

## Conflicts of interest

There are no conflicts to declare.

## Supplementary Material

SC-014-D3SC02629A-s001
