# Supplementary material for: Feshbach resonances in the F + CHD3 → HF + CD3 reaction
Source: Chem Sci. 2023 Jun 24;14(29):7973–9. doi: 10.1039/d3sc02629a (PMC10370578; doi:10.1039/d3sc02629a)
Supplement: SC-014-D3SC02629A-s001 [file SC-014-D3SC02629A-s001.pdf]

Electronic Supplementary Material (ESI) for Chemical Science.
This journal is © The Royal Society of Chemistry 2023

Supplementary Information

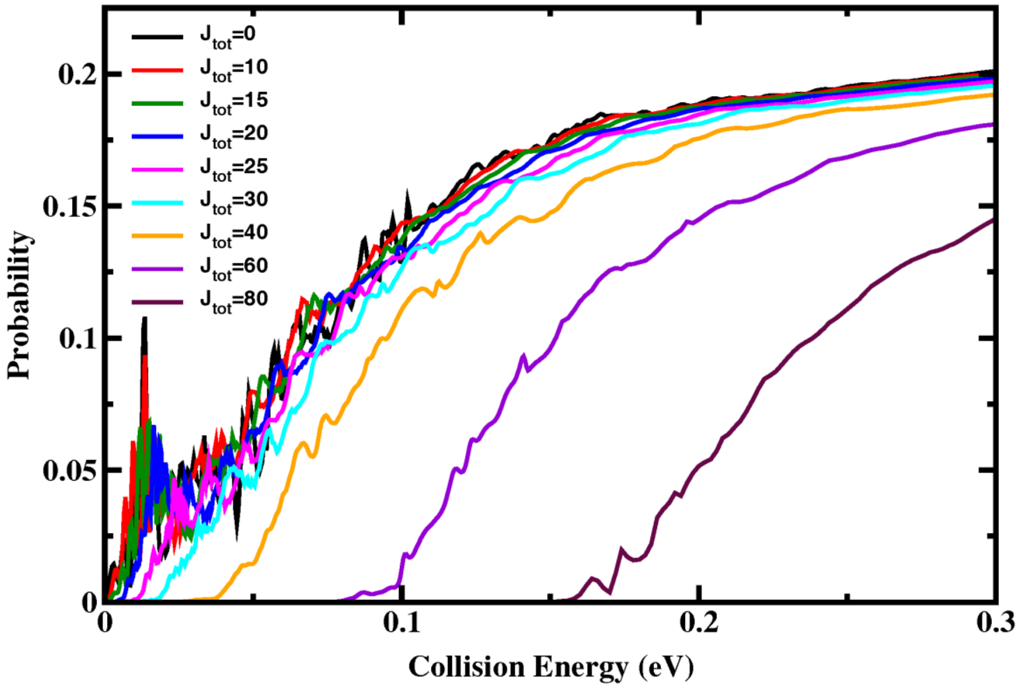

Fig. S1 The total reaction probabilities for the total angular momentum J_{tot}=0, 10, 15, 20, 25, 30, 40, 60, and 80 for the initial ground rovibrational state as a function of collision energy.

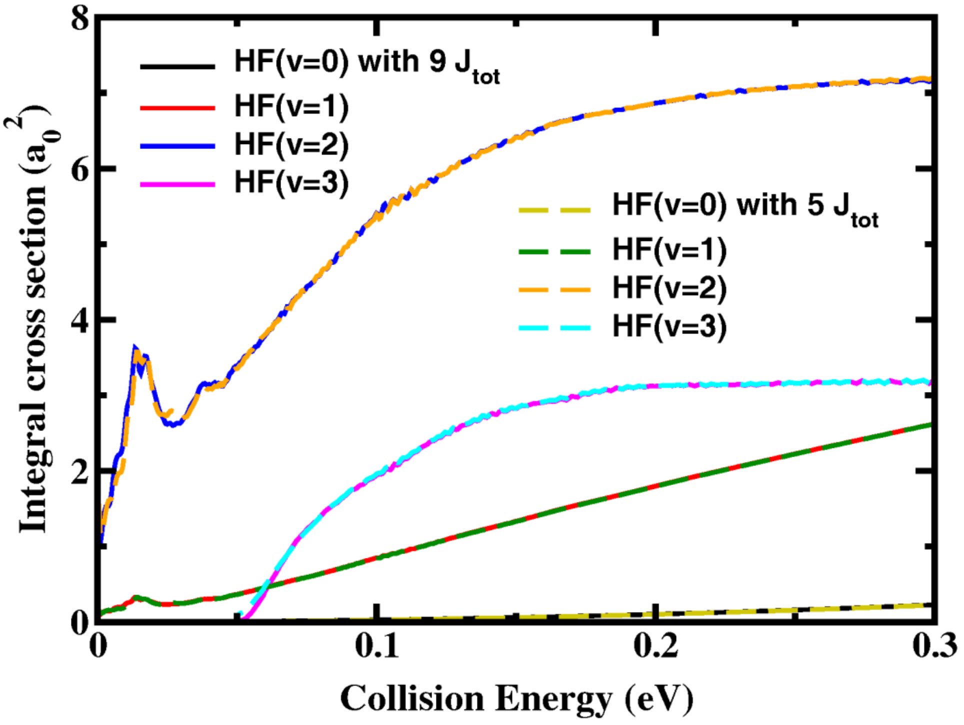

Fig. S2 Product HF vibrational state-resolved ICS as a function of collision energy calculated with 9 J_{tot} (0, 10, 15, 20, 25, 30, 40, 60, 80), in comparison with those calculated with only 5 J_{tot} (0, 20, 40, 60, 80).

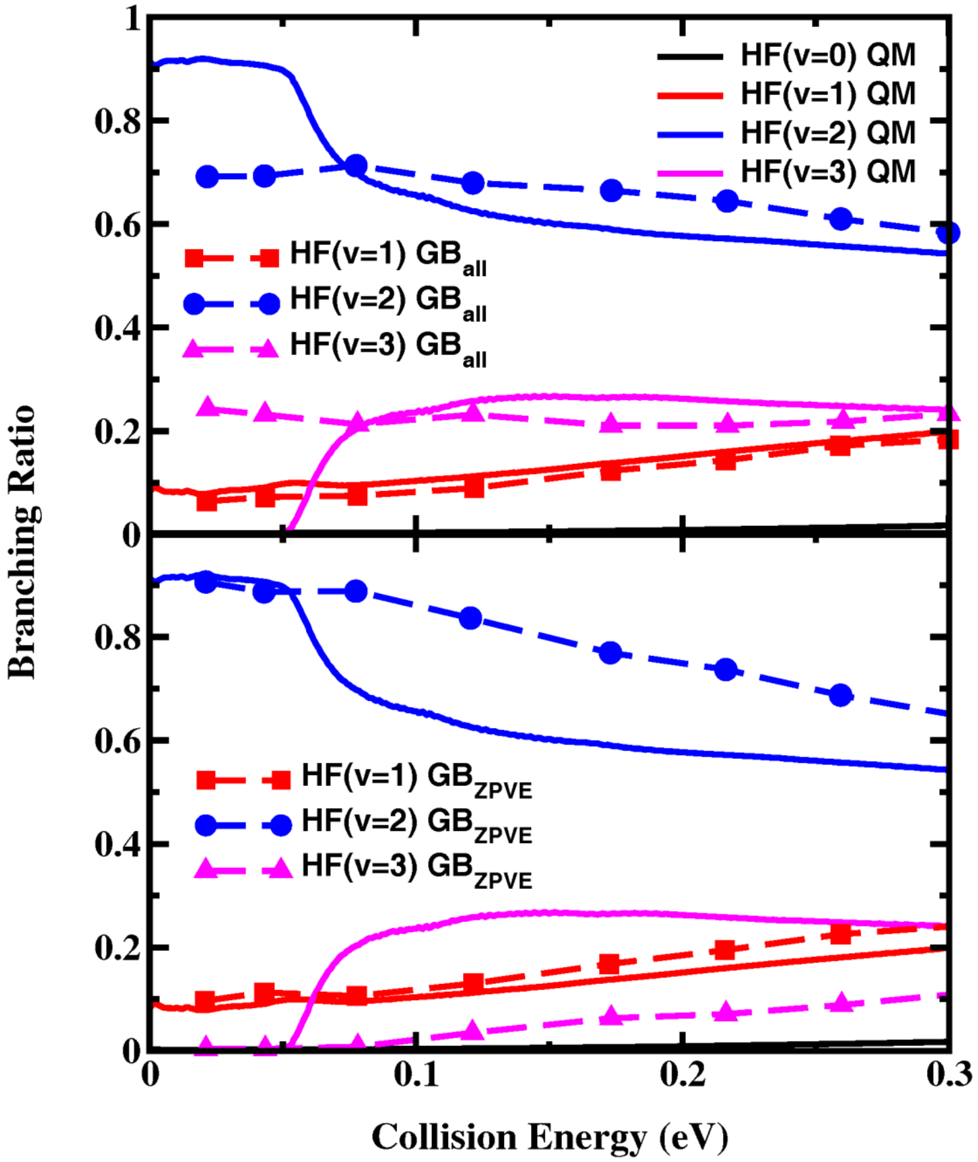

Fig. S3 Normalized HF vibrational populations as a function of collision energy for the title reaction, in comparison with the QCT results in Ref 45.

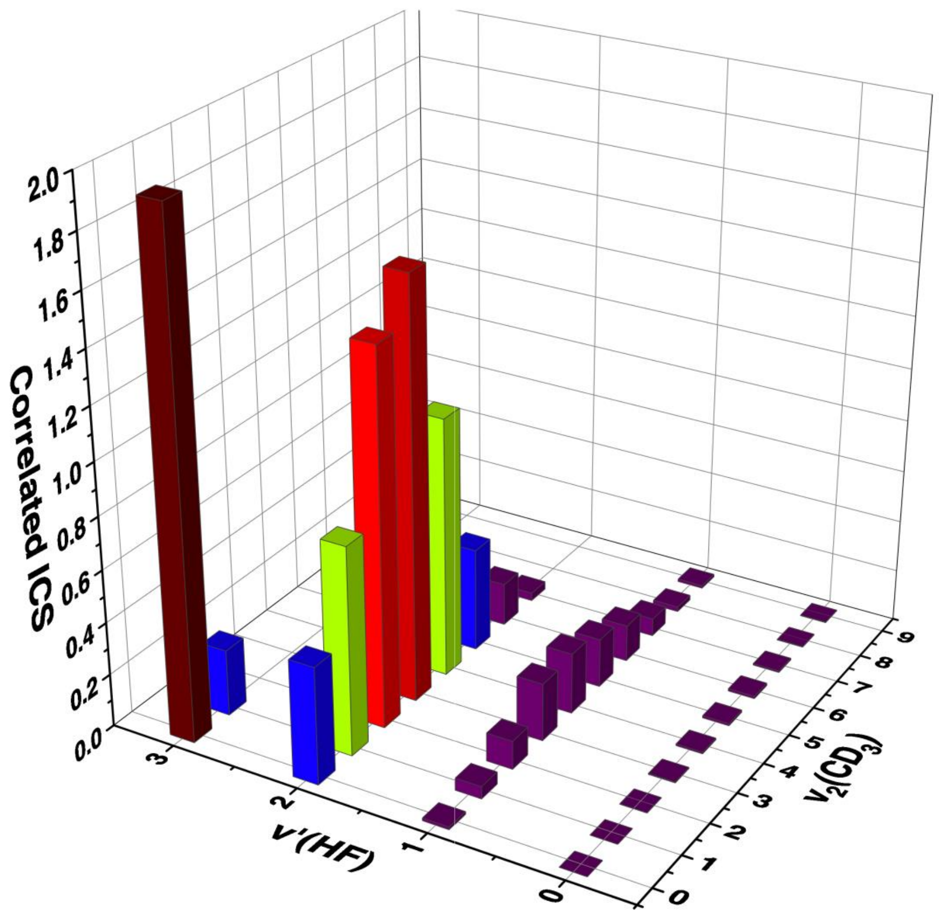

Fig. S4 3-dimensional representation of the correlated ICS of the product pair of HF(v') and CD_3(v_2) at the collision energy of 0.121 eV.

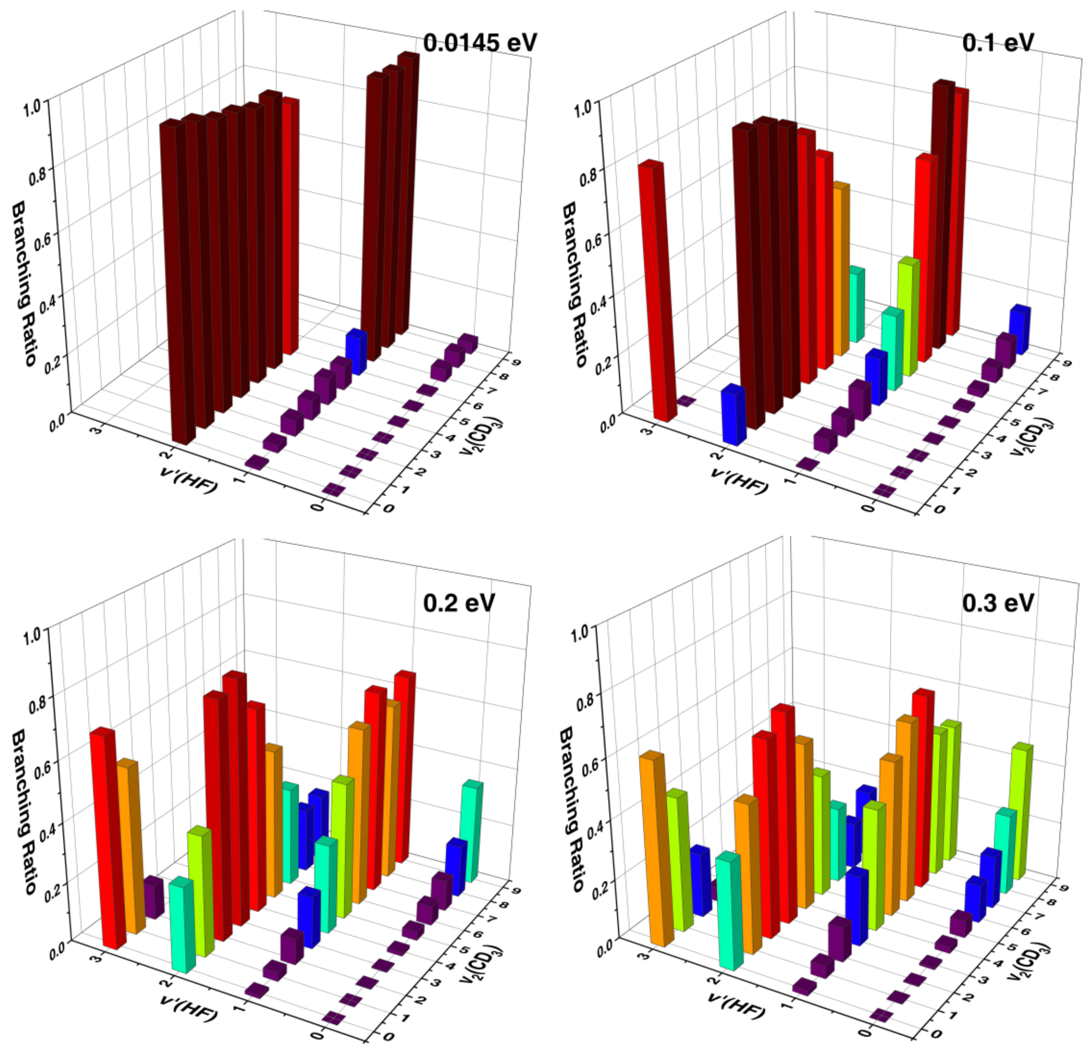

Fig. S5 3-dimensional representation of the correlated ICS of the product pair of HF(v') and $CD_3(v_2)$ at the collision energy of 0.0145 eV, 0.1 eV, 0.2 eV and 0.3 eV, that the sum of correlated HF vibration populations for each CD_3 state at a given E_c are scaled to unity.

Table S1. Energy disposal (in eV) into $HF(v')+CD_3(v_2)$ products when HF vibrationally excited from v'=0 to 3.

HF state	v'=0	v'=1	v'=2	v'=3
$E_{HF}(v')$	0	0.491	0.96	1.409
E_c=0.0145 eV				
$\langle E_{v2}\rangle_{CD3}$	0.399	0.298	0.2	–
$\langle E_{v2}\rangle_{CD3}+E_{HF}(v')$	0.399	0.789	1.16	–
E_c=0.1 eV				
$\langle E_{v2}\rangle_{CD3}$	0.368	0.276	0.171	–
$\langle E_{v2}\rangle_{CD3}+E_{HF}(v')$	0.368	0.767	1.132	–
E_c=0.2 eV				
$\langle E_{v2}\rangle_{CD3}$	0.364	0.263	0.153	0.036
$\langle E_{v2}\rangle_{CD3}+E_{HF}(v')$	0.364	0.754	1.114	1.445
E_c=0.3 eV				
$\langle E_{v2}\rangle_{CD3}$	0.359	0.25	0.143	0.049
$\langle E_{v2}\rangle_{CD3}+E_{HF}(v')$	0.359	0.741	1.104	1.458

Table S2. Energy disposal (in eV) into HF(v')+CD_3(v_2) products when CD_3 vibrationally excited from v_2=0 to 9.

CD_3 state	v_2=0	v_2=1	v_2=2	v_2=3	v_2=4	v_2=5	v_2=6	v_2=7	v_2=8	v_2=9
$E_{CD3}(v_2)$	0	0.056	0.119	0.186	0.257	0.331	0.407	0.486	0.567	0.649
E_c=0.0145 eV										
$<E_v>_{HF}$	0.956	0.947	0.935	0.929	0.917	0.919	0.892	0.469	0.465	0.471
$<E_v>_{HF}+ E_{CD3}(v_2)$	0.956	1.004	1.054	1.115	1.174	1.25	1.3	0.955	1.031	1.12
E_c=0.1 eV										
$<E_v>_{HF}$	1.328	0.937	0.929	0.908	0.879	0.827	0.755	0.583	0.443	0.416
$<E_v>_{HF}+ E_{CD3}(v_2)$	1.328	0.993	1.048	1.095	1.136	1.158	1.162	1.069	1.01	1.065
E_c=0.2 eV										
$<E_v>_{HF}$	1.27	1.198	0.971	0.873	0.808	0.715	0.616	0.542	0.509	0.324
$<E_v>_{HF}+ E_{CD3}(v_2)$	1.27	1.254	1.09	1.059	1.065	1.046	1.023	1.028	1.076	0.973
E_c=0.3 eV										
$<E_v>_{HF}$	1.23	1.143	1	0.864	0.745	0.657	0.549	0.477	0.462	0.292
$<E_v>_{HF}+ E_{CD3}(v_2)$	1.23	1.199	1.119	1.05	1.003	0.989	0.956	0.963	1.029	0.941

A. Numerical parameters

The numerical parameters used in reactant Jacobi coordinates are as following: A total number of 280 sine basis functions covering a range from 3.0 to 19.8 bohrs were used for R with 140 grid points in the interaction region. For the r dimension, 100 basis functions were used in the range of [1.0,11.0] bohrs in the interaction region, while 6 basis functions were used in the asymptotic region. The number of basis functions for the umbrella motion was 16. The rotational basis functions were constrained by the parameters, J_{max}=204, l_{max}=180, j_{max}=24. The center of the prepared Gaussian wave packet was located at R_0=18.0 bohrs, with the width of δ=0.2 bohr, and the central energy of E_0=0.15 eV. For total angular momentum J_{tot}=0, we propagated the wave packets for 150,000 a.u. of time with a time step of 10 to converge the reaction probabilities. The absorption potential on the r coordinate for the MRPD calculation takes the form

$$V_p(r) = C(\frac{r-r^0}{\Delta r})^n,$$

with r^0=9.0 a.u., Δr=2.0 a.u., C=0.04, and n=1.5. To minimize the computational cost, the coordinate transformation is carried out at every 8 propagation time steps. Continuous propagation in the product Jacobi coordinates only involves a total number of 300 sine functions for the translational coordinate in a range of [3.0,21.0] bohrs and 7 vibrational basis functions for HF bond. The number of basis functions for the umbrella motion was 11. The rotational basis functions were constrained by the parameters, J'_{max}=54, l'_{max}=30, and j'_{max}=24. A dividing surface is placed at R'=15.5 bohr to extract S matrix elements. The number of K'-blocks included in the product calculation increases with the total angular momentum, from 1 for J_{tot}=0, up to 6 for J_{tot}=80.
